# Multi-TGDR: A Regularization Method for Multi-Class Classification in Microarray Experiments

**DOI:** 10.1371/journal.pone.0078302

**Published:** 2013-11-19

**Authors:** Suyan Tian, Mayte Suárez-Fariñas

**Affiliations:** 1 Division of Clinical Epidemiology, First Hospital of the Jilin University, Changchun, Jilin, China; 2 Center for Clinical and Translational Science, The Rockefeller University, New York, New York, United States of America; 3 Laboratory for Investigative Dermatology, The Rockefeller University, New York, New York, United States of America; University of Georgia, United States of America

## Abstract

**Background:**

As microarray technology has become mature and popular, the selection and use of a small number of relevant genes for accurate classification of samples has arisen as a hot topic in the circles of biostatistics and bioinformatics. However, most of the developed algorithms lack the ability to handle multiple classes, arguably a common application. Here, we propose an extension to an existing regularization algorithm, called Threshold Gradient Descent Regularization (TGDR), to specifically tackle multi-class classification of microarray data. When there are several microarray experiments addressing the same/similar objectives, one option is to use a meta-analysis version of TGDR (Meta-TGDR), which considers the classification task as a combination of classifiers with the same structure/model while allowing the parameters to vary across studies. However, the original Meta-TGDR extension did not offer a solution to the prediction on independent samples. Here, we propose an explicit method to estimate the overall coefficients of the biomarkers selected by Meta-TGDR. This extension permits broader applicability and allows a comparison between the predictive performance of Meta-TGDR and TGDR using an independent testing set.

**Results:**

Using real-world applications, we demonstrated the proposed multi-TGDR framework works well and the number of selected genes is less than the sum of all individualized binary TGDRs. Additionally, Meta-TGDR and TGDR on the batch-effect adjusted pooled data approximately provided same results. By adding Bagging procedure in each application, the stability and good predictive performance are warranted.

**Conclusions:**

Compared with Meta-TGDR, TGDR is less computing time intensive, and requires no samples of all classes in each study. On the adjusted data, it has approximate same predictive performance with Meta-TGDR. Thus, it is highly recommended.

## Introduction

Biomarker discovery from high-dimensional data is a crucial problem with enormous applications in areas of biomedical research and translational medicine. Selecting a small number of relevant features (e.g., genes in transcriptomics profiles, SNPs in GWAs studies, and metabolites in metabolomics) to build a predictive model that can accurately classify samples by their diagnosis (e.g., diseased or health, different stages of one specific cancer) and prognosis (e.g., potential response to a given treatment, 5-year survival with a certain treatment) is an essential step towards personalized medicine. In bioinformatics, such a task is accomplished by a feature selection algorithm, which besides reducing over-fitting and improving classification accuracy, leads to small molecular signatures with manageable experimental verification and the potential design of cheap dedicated diagnostic and prognostic tools.

Among dozens to hundreds of proposed feature selection algorithms [1]–[3], the Threshold Gradient Decent Regularization (TGDR), proposed by Friedman and Popescu [4], stands out because of the elegant theory beneath them, its easy-to-moderate programming difficulty for a well-trained statistician and its good performance and biologically meaningful results in real-world applications. TGDR builds upon the classical gradient descent by introducing a threshold parameter that directs the paths towards a parameter with more diverse components. Ma and Huang [5] elegantly extended the TGDR to the case where expression data from several studies are combined. The proposed algorithm, the Meta Threshold Gradient Descent Regularization (Meta-TGDR), assumes that the same set of genes is selected on all studies, while allowing the β coefficients to vary across studies, in a meta-analysis fashion. In their paper, they demonstrated that a better classification performance was achieved by using Meta-TGDR rather than by using TGDR on the combined data set.

However, both TGDR as originally proposed, and Meta-TGDR frameworks do not give the explicit definition or/and format on the multi-class classification where an observation needs be categorized into more than two classes. Additionally, Meta-TGDR [5] does not offer an overall predictive rule on an independent data set (testing samples), from a study not used in classifier training/estimation. The absence of such rule prevented Meta-TGDR from the evaluation of its performance on independent testing sets, and the comparison with TGDR in terms of predictive performance. Furthermore, it limited the use of the Meta-TGDR to real, clinical practice application precluding its use in personalized medicine, where a classifier is built for use in an extended population under variable laboratory setting.

In this paper, we specifically addressed the first issue by proposing a new framework, referred as to multi-TGDR, and the second issue by proposing an equation. Lastly, the results from Meta-TGDR, and TGDR on the pooled data were compared in terms of their predictive performance.

## Materials and Methods

### The proposed extensions to TGDR and meta-TGDR

In order to establish the nomenclature to be used through the paper and to help the reader experience we start with a brief description of the Meta-TGDR as below. The interested readers are referred to [5] for more details on both TGDR and Meta-TGDR frameworks.

Let 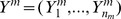 be the indicator function (i.e., 0 if in the reference class, 1 otherwise) for each study m = 1,.,M with n_m_ subjects and 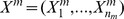 the vector of n_m_xD matrices representing the gene expression for each subject over the same set of D genes. The likelihood function for study *m* can be written as:
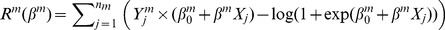
(1)where 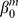 and 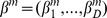 are the unknown intercept and expression-coefficients for each study s. These parameters will be simultaneously estimated by maximizing the overall likelihood function R(β) = R^1^(β^1^)+…+R^M^(β^M^) with 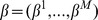
where only the coefficients associated with gene expression are subject to regularization,

Denote Δ*v* as the small positive increment (e.g., 0.01) as in ordinary gradient descent searching, *v_k_* = k×Δ*v* the index for the point along the parameter path after *k* steps; and β(*v_k_*) the parameter estimate of β corresponding to this point. For a given fixed threshold 0*≤τ≤*1, the Meta-TGDR algorithm iterate on the following steps:

1. Initialize β(0)  = 0 and *v_0_* = 0. (i.e., all genes are non-informative).
2. With current estimate β,
  - Compute the negative gradient matrix 
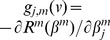
.
  - Define the meta-gradient G (v) as a D-dimensional vector whose j^th^-element is the sum of the gradient for each study; i.e., 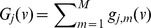.
  - if 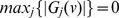, stop the iteration.
3. Compute the threshold vector *f(v)* of length *D*, where the j^th^ component of *f(v)*:
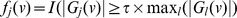

4. Update β(v+Δv) =  β(v) – Δv×g (v) ×f(v) and update *v* by *v*+ Δv, where the product of *f* and *g* is component-wise.
5. Steps 2–4 are iterated *k* times. Both τ and *k* are tuning parameters and determined by cross validation.

In both TGDR and Meta-TGDR, the search path is determined by the gradient g (i.e., both the direction and magnitude), and the threshold vector f(v) that establish which features are selected through the tuning parameter τ. Uniquely to the Meta-TGDR, the concept of meta-gradient G, defined as the sum of each study's negative gradient, was introduced to define the direction of the search path. In each iteration, features with the largest meta-gradient would be selected, with the number of features being regularized by τ. If τ = 0, all features are included in the model whereas a τ = 1 implies that only the feature with the largest meta-gradient is selected. By this way, Meta-TGDR selects the same set of features, but their corresponding coefficients may differ across studies.

#### Multi-TGDR: Extension to multi-class classification

Multiple-class classification is commonly encountered in real world, however, many proposed feature selection algorithms lack the valuable capacity of dealing with multi-class classification. In the original TGDR and Meta-TGDR framework, multi-class classification had been left untouched even though all authors claimed that such extension is very natural. Here, we propose an extension of the TGDR framework to multi-class cases.

In the multi-class scenario, the response variable Y_i_ -representing the class membership for subject *i* – may take values 1,…,K, where *K* is the number of classes (K≥3). Propositions to tackle this problem using existing binary classifiers divided into two major types: “one-versus-the-rest” where K binary classifiers were trained to distinguish the samples in a single class from the samples in all remaining classes, and “one-versus-another” where K(K-1)/2 classifiers were trained to distinguish the samples in a class from the samples in one remaining class. Many researchers [6]–[8] had demonstrated that one-versus-another schema offered better performance than one-versus-the-rest did. Therefore, we compared our proposed framework with one-versus-another schema only.

The central idea of our extension is to replace the single indicator variable Y_i_ for each sample for a set of K-1 variables Y_ik_. The threshold function is then defined as the maximum along the set of local threshold functions, defined on the subspaces defined by parameters associated to each class. To our knowledge, multi-class TGDR has not been addressed; probably because it is more computationally demanding than binary TGDR.

Let Y_k1_,…,Y_kn_ be the vector of indicators for class *k* across subjects; i.e., Y_kj_ is equal to 1 if the j^th^ subject belongs to class k and zero otherwise. This vector is defined for each class k(k = 1,…,K-1), while the K^th^-class serves as the reference class. In order to make our classes mutually exclusive,
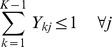



As before, let X_1_,…,X_n_ represent the gene expression values. After a simple algebraic manipulation,the log-likelihood function can be written as:
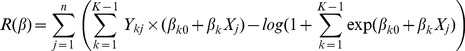
(2)


β_k0_'s are unknown intercepts which would be not subject to regularization. β_k_ =  (β_k1_,…, β_kD_) are the corresponding coefficients for expression values for the same set of D genes for the comparison between class k and reference class K. Note, the dimension of all β_k_ is restricted to be the same, but their magnitudes differ. Hence the same number of non-zero genes is used on all classes but their estimated values are different.

Let 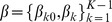 denote the set of all parameters to be estimated in model 3, one can follow the binary TGDR procedure as detailed in [5], [9], [10] but introducing the following modification in the calculation of the threshold vector *f(v)* in step 3:

Here, 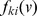 represents the threshold vector of size D for class k(k = 1,.,K-1),
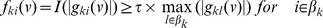



Then, the *i^th^*-gene specific element of threshold function f(v) will be obtained as:
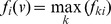



As in binary TGDR and Meta-TGDR, all genes are assumed to be non-informative at the initial stage. The tuning parameters, τ and k jointly determine the property of the estimated coefficients (i.e., which ones would be selected, and their corresponding magnitudes). When τ = 0, all coefficients are nonzero for all values of k. When τ = 1, the multi-TGDR usually increases in the direction of feature (i.e., gene here) with the largest gradient in each iteration and the non-zero coefficients are few for a relatively large number of iterations. Since by definition f(v) is 1 as long as one of the elements 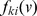 is 1, it indicates that if a feature is selected because is important for, let's say, class 1 (versus reference class K), then it would appear in all the other comparisons even though its corresponding coefficients in those comparisons may not differ significantly from zero.

Here we establish a unique τ tuning parameter, this assumption may be relaxed so that τ can have different values for each class, which will allow different degree of regularization for different comparisons. The proposed framework is referred to as *multi-TGDR*.

#### Multi-class Meta-TGDR

The Multi-TDGR can naturally be extended to a situation with multiples studies and multiple classes. The step 3 in multi-class TGDR is combined with the concept of meta-gradient of Meta-TGDR. That means *f_ki_* is defined on the meta-gradient instead of the regular gradient, i.e., 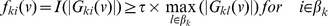
and 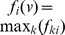. Under this formulation, the same set of genes is selected for each class and each study but coefficients can vary across studies and classes. Obviously, with the increase on β's dimension, the iterations are more time-consuming compared to multi-class TGDR. In this paper, this framework is referred as to *Meta-multi-TGDR.*

#### Prediction of new samples using Meta-TGDR

In the Meta-TGDR, the estimated coefficients β =  (β_1_,…,β_D_), corresponding to expression values for D genes selected, are different in each study. This raises a question of how to use these study-specific coefficients to perform an overall prediction in a new independent sample (“testing sample”), not previously used in the training/estimation stage. However Ma and Huang [5] did not offer a solution to this issue, which preclude the evaluation of the performance of the Meta-TGDR (and its comparisons with TGDR) on independent “testing” samples. Here we extended their work by conducting a meta-regression to synthesize the results from Meta-TGDR and extrapolate the membership prediction to a sample from a new study. Under the Meta-TGDR settings, let *Z_ij_* be the estimated log odds for the *j* ^th^ sample in *i* ^th^ study using the estimated coefficients 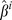 (of length D) for each study (i.e., 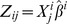). Z_ij_ can be modeled as:
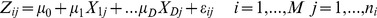
(3)where μ_1_,…, μ_D_ represents the overall coefficient associated with gene i, 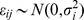 and 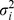 is the within-study variance for the study *i*. Specification of equation 3 can be achieved by following a 3-step procedure with the first two steps obtain estimates of 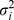
using delta method [11] and step 3 calculates the posterior probabilities using the estimated coefficients obtained in step 2.

1 Consider 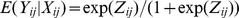, and 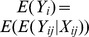. For each study estimate the variance of Y in natural scale by:
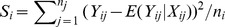

2 Estimate 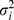 using delta-method i.e.,
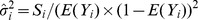

Once we have 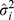, the overall estimated coefficients μs can be easily obtained by a weighted least square equation (similar to the one used in fixed-effect meta-analysis).

3 Finally, the overall μ_i_'s estimated in step 2 is used to calculate the odd-ratio and the posterior class-membership probability for a new sample.

### Miscellaneous

#### Stabilization of the selected genes using Bootstrap aggregating

In order to improve the stability and classification accuracy of multi-class TGDR, we applied Bootstrap aggregating (Bagging) to our classifier [12]. Given a training set of size n, bagging generates *m* new training sets, each of size n, by sampling subjects from the original training set with replacement. Then *m* multi-class TDGRs are conducted using the above *m* bootstrap samples and combined by voting. Bagging helps to protect over-fitting which usually exists in the classification setting.

#### Evaluation of predictive performance

The performance of a classifier is measured using traditional performance metrics over the training samples (training: # of misclassification/sample size on training set; and 5 fold-cross-validation misclassified error rate: the misclassified rate on the cross-validation data) and over the test samples (the predictive error, which is more heavily weighted than the misclassification errors when we evaluate the predictive performance of an algorithm since those samples were not used to construct the classifiers, thus less subject to over-fitting). Since the membership probabilities for each sample can easily be obtained from multi-TGDR/TGDR algorithm, we also used the generalized Brier score (GBS) proposed by Yeung et al [13], a generalization of the Brier Score to a multi-class classification problem, to evaluate the performance of these algorithms (i.e., multi-TGDR, TGDR and its pairwise coupling, and Meta-TGDR). Under the K class setting, where *Y_ik_* are indicator functions of class *k *(*k* = 1,…,*K*), let 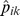
denote the predicted probability such that Y_ik_ = 1. For easier interpretation and comparison of GBS score across different classification settings, we normalized the GBS by the sample size n as in [14] i.e., 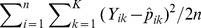. By taking into consideration the magnitudes of predicted probabilities, GBS, can establish difference in performance of classifiers with same overall predictive error. The smaller the GBS value, which after normalization takes values in [0,1], the better a classifier performs.

#### The Experimental Data and preprocessing procedures

**Psoriasis:** Open-access data from 3 published studies [15]–[17] available under GEO accession numbers GSE14905, GSE13355, and GSE30999, respectively were used, including samples from Lesions (LS) and adjacent Non-Lesional (NL) skin form psoriasis patients and Normal skin from healthy patients. Details of these studies – using Hgu133plus2 Affymetrix chips- are given in [18].

**Lung cancer:** The lung cancer data sets included GSE10245, GSE18842, and GSE2109, all studies were performed on Affy HGU 133plus2 chips and publicly available on the GEO repository.

**Pre-processing procedures:** The raw Affymetrix data (CEL files) of both lung cancer and psoriasis data sets were downloaded from GEO repository and expression values were obtained using **FRMA** algorithm [19] [20]. To pool data from different studies together and to address the batch effects from different experiments, COMBAT algorithm [20] was used to adjust on the combined expression values for these two combined data sets. For the lung cancer data, moderated F/t-tests (limma package) were conducted to identify differentially expressed genes (DEGs) with cutoffs for FDR and fold change as 0.05 and 2, respectively. When there are multiple probesets representing the same gene, the one with the largest F-value was chosen. The resulting 949 unique genes were fed into the downstream analysis. Note, for the TGDR algorithm there is no limit on the number of genes fed into the algorithm. However, it is common practice in high-throughput experiments (e.g., Ma and Huang [5], [9]) to rule out “non-informative” genes using a filtering procedure before the classification. By doing so, a large amount of computing time can be saved with only partial set of genes put in classifiers; but with no or least loss on the potential biomarkers since almost all genes which have high probability to be biomarkers pass the filtering. Relevantly, Tritchler et al. [21] explored the effect of filtering on some downstream analysis (i.e., clustering and network analysis) in their work and discussed in details the advantages of filtering as a preprocessing step. For psoriasis data, the filtering steps taken (including SD, ICC and DEGs using meta-analysis method) were used by us previously and described in details there [18]. Similar to the lung cancer data, conducting those filtering steps is mainly for the purpose of saving the computing time. 2301 unique genes passed the filtering were fed into multi-TGDR and Meta-TGDR algorithms.

#### Statistical language and packages

The statistical analysis was carried out in the R language version 2.15 (www.r-project.org), and packages were from the Bioconductor project (www.bioconductor.org). R code for multi-TGDR is available upon request.

## Results and Conclusions

### Simulation studies

In this section, we use two simulated examples to study the empirical performance of multi-TGDR.

### Example 1

In the first simulation, 100×n iid standard normal (mean  = 0, variance  = 1) random variables (i.e., X_1_,…,X_100_, those are vectors of length n, n is the sample size), and n class membership outcome variables (Y_1_,…,Y_n_) taking the values of 1–3 were simulated. The logit function for class 2 and 3, having class 1 as reference, was calculated through the following relationship:
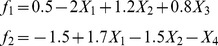
where the logit for class 2 depends only on features X_1_ X_2_ X_3_ and class 3 logit depends on features 1,2 and 4. According to this model, 50 data sets were generated and analyzed by the proposed multi-TGDR framework. Results for this simulation, summarized in Table 1A shows that almost 100% of times the relevant features were selected by multi-TGDR framework. As criticized by Wang et al [22], lack of parsimony is an obvious disadvantage of TGDR algorithms, a shortcoming inherited by the multi-TGDR. However, the introduction of the Bagging procedure improves upon parsimony.

**Table 1:** The results for simulated data.

A. Simulation 1 (the whole data)						
	% of β_1_≠0 Average BF (%)	% of β_2_≠0 BF (%)	% of β_3_≠0 BF (%)	% of β_4_≠0 BF (%)	Average # of selected genes	Average predictive error (%)
Multi-TGDR	100	100	100	98	28.2	13.90
without bagging	**100**	**99.98**	**91.48**	**90.64**		
Multi-TGDR BF>40%	–	–	–	–	21	13.98
BF>80%	–	–	–	–	5.42	11.96
On the reduced data, where top 20 features were kept						
Multi-TGDR	100	100	92	98	18.4	15.82
no bagging	**100**	**100**	**91.60**	**97.68**		
BF>40%	–	–	–	–	3.9	10.00
BF>80%	–	–	–	–	3.9	10.00
B. Simulation 2 (the whole data)						
Multi-TGDR	100	100	96	100	30.2	13.26
without bagging	**100**	**100**	**90.88**	**95.48**		
Multi-TGDR BF>40%	–	–	–	–	22.62	13.20
BF>80%	–	–	–	–	5.54	11.08
On the reduced data, where top 20 features were kept						
Multi-TGDR	100	100	82	94	18.2	15.22
No bagging	**100**	**100**	**81.60**	**93.00**		
BF>40%	–	–	–	–	5.5	9.24
BF>80%	–	–	–	–	3.76	9.24

### Example 2

To explore the effect of the correlations among features (i.e., independent variables) may have on multi-TGDR, we set the simulations as in the previous example but assumed the following correlations among features: cor (X_1_, X_5_)  =  cor (X_3_, X_7_)  = 0.8 and cor (X_2_, X_6_)  =  cor (X_4_, X_8_)  = −0.8. Table 1B presents the results for this simulation. When compared with the uncorrelated scenario of example 1, the size of final selected feature is marginally larger while the predictive errors are almost the same. Nevertheless, multi-TGDR always selected the relevant features successfully and has good predictive performance. Bagging procedure improves upon both parsimony and predictive performance. Thus, it is highly recommended to combine bagging with any TGDR algorithm although bagging is very computing-time intensive.

In high-throughput experiments, it is common practice to use low-level analysis to eliminate non-informative features before embarking on more complex, time-consuming modeling. To evaluate if the pre-processing steps used in our data (see methods section) can erroneously filter out the informative features, we reran both simulations using only the 20% top features (ranked by *limma*'s moderated t-tests). The results show that the exclusion of non-informative features deemed by a pre-processing filtering would not degrade the predictive performance of the final models. In terms of implementation on real-world applications, this filtering step eases the computational burden of downstream analysis (i.e., TGDR and Bagging) by reducing the initial dimensions of 50 k to a few thousands; a reasonable size even for a PC.

### Applications on microarray expression data

Using the real-world applications, the appropriateness and accuracy of the proposed multi-TGDR was evaluated. We also compared the performance of Meta-TGDR and TGDR under a priori batch-adjustment.

### Lung cancer

About 80% of lung cancers (LC), the leading cause of cancer-related death throughout the world, are classified as non-small cell lung carcinoma (NSCLC) with Adenocarcinoma (AC) and squamous cell carcinoma (SCC) the two major subtypes of NSCLC. SCC is characterized as a poorly differentiated tumor subtype that develops in the proximal airways and is strongly associated with cigarette smoking. In contrast, AC usually arises in the peripheral airways and is more commonly observed in non-smokers and women. Mutations have been identified in AC and not in SCC, suggestion different mechanism of progression and treatment response.

For LC data, we randomly divided it into 4 subsets with roughly equal sizes and used 3 fold of them as the training set (n = 109) and the remained 1-fold as the test set (n = 36). Using multi- TGDR algorithm, 67 biomarkers were identified with 0% training error and a predictive error of 20.2% in a 5-fold cross-validation (CV). The comparison between pair-wise TGDRs and the multi-class TGDR is summarized in Table 2, and it shows that multi-TGDR over-perform the pair-wise strategy in all performance statistics. In terms of computing time, a single run of binary TGDR (including the determination of tuning parameters using cross-validation and model estimation) took on average 19 seconds on a MacBook equipped with double-core 1.8 GHz processors and 8GB RAM. Thus, for the LC data, the total computing time for the pair-wise TGDRs was about 2 minutes and only 77 seconds for the multi-TGDR framework, saving about 36% of the computing time.

**Table 2:** Performance of classifiers for Lung Cancer data.

		Training (N = 109)			Test set (n = 36)	
		# genes	Error (%)	CV (%)	Predictive Error (%)	GBS
Pair-wise	ACI vs ACII	13	12.77	29.78		
	ACI vs SCC I	28	0	11.63		
	ACI vs SCC II	19	0	12.00		
	ACII vs SCCI	15	0	3.39		
	ACII vs SCCII	13	0	8.70		
	SCCI vs SCC II	44	22.58	35.48		
	***Overall***	***107***	***18.34***	***51.38***	***50.00***	***0.302***
Multi-TDGR	*No Bagging*	***67***	***0***	***20.2%***	***47.22***	***0.292***
	with Bagging	19	9.17	–	44.44	0.303

After applying Bagging (N_B_ = 100) to the LC data, we found that all genes in the 67-gene signature produced by multi-TGDR appear in the classifier with more than 5% frequency and 19 of them has bragging frequencies (BF) larger than 40% (Table 3). CYP24A1 is the gene most frequently selected (77%) followed by PNLDC1 (67%). By reducing the multi-TDGR signature to those genes with BF >40%, the performance showed slightly improvement by a predictive error reduction of 2.78% on the test set.

**Table 3:** Multi-TGDR genes for lung cancer data after Bagging.

Probe	Symbol	Description	βAC-II	βSCC-I	βSCC-2	Freq
206504_at	CYP24A1	cytochrome P450, family 24, subfamily A, polypeptide 1	0.2931	−0.3501	0.0692	0.77
1564414_a_at	PNLDC1	poly(A)-specific ribonuclease (PARN)-like domain containing 1	−0.0499	−0.0942	0.3569	0.67
211416_x_at	GGTLC1	gamma-glutamyltransferase light chain 1	−0.2747	−0.1519	−0.1726	0.65
206059_at	ZNF91	zinc finger protein 91	−0.0755	0.0557	−0.2927	0.55
205348_s_at	DYNC1I1	dynein, cytoplasmic 1, intermediate chain 1	0.1083	0.0092	0.388	0.54
231867_at	ODZ2	odz, odd Oz/ten-m homolog 2 (Drosophila)	0.0065	0.2616	−0.0077	0.51
219926_at	POPDC3	popeye domain containing 3	0.4104	0.1598	0.1239	0.51
219298_at	ECHDC3	enoyl CoA hydratase domain containing 3	−0.0718	0.0835	−0.3062	0.5
203358_s_at	EZH2	enhancer of zeste homolog 2 (Drosophila)	−0.5307	−0.0307	0.4366	0.49
214464_at	CDC42BPA	CDC42 binding protein kinase alpha (DMPK-like)	−0.0406	−0.3358	−0.1537	0.48
210020_x_at	CALML3	calmodulin-like 3	−0.0172	0.2187	0.0875	0.46
201839_s_at	EPCAM	epithelial cell adhesion molecule	−0.0045	−0.0408	−0.0017	0.45
238983_at	NSUN7	NOP2/Sun domain family, member 7	0.0135	−0.2718	−0.019	0.45
206677_at	KRT31	keratin 31	0.0076	−0.0138	0.1216	0.44
235706_at	CPM	carboxypeptidase M	0.1549	−0.0625	0.0164	0.43
226213_at	ERBB3	v-erb-b2 erythroblastic leukemia viral oncogene homolog 3 (avian)	−0.0138	−0.1827	−0.0757	0.43
205713_s_at	COMP	cartilage oligomeric matrix protein	0.0632	−0.2135	0.048	0.41
228846_at	MXD1	MAX dimerization protein 1	0.026	0.0184	0.0838	0.41
227492_at	OCLN	Occluding	0.0015	−0.4148	−0.1417	0.41

Here, AC-I serves as the reference. Bagging frequency >40%.

We concluded that bagging procedure discarded the random noises produced by a single run of TGDR. Furthermore, the calculation of membership probabilities in multi-class TGDR is more straightforward compared to the pair-wise coupling. Given it is intrinsically challenging to derive meaningful diagnostic signatures from high-throughput experiments in complicated problems like this, one major objective of presenting this data set is to use it as a benchmark for the development of more suitable classifiers on lung cancer subtypes and stages.

### Psoriasis

Psoriasis vulgaris is a common chronic inflammatory skin disease of varying severity, characterized by red scaly plaques. Publicly data from 3 published studies [15]–[17] were used, including samples from Lesional (LS) and adjacent Non-Lesional (NL) skin of psoriasis patients and Normal skin from healthy patients.

Here, we used the psoriasis data to investigate the effect of the Batch/study adjustment in the performance of TGDR and Meta-TGDR, as well as to further evaluate multi-TGDR. Again, we randomly divided the whole data into 5 subsets with roughly equal sizes and used 4 fold of them as the training set (n = 360) and the left one fold as one test set (n = 89). By doing so, we can evaluate the validity of the proposed equation for the overall estimates since both the study-specific and overall estimates are available for the samples in this test set.

Here, we first present the performance of the binary classifiers followed by the multi-class problem. The binary classifiers (LS vs Normal, LS vs NL and NL vs Normal) will allow us 1) to assess the effect of batch adjustment on the performance of TGDR and Meta-TGDR and 2) to evaluate the validity of the method proposed here to allow prediction of independent data set in the Meta-TDGR framework.

#### Binary Classification Problems in Psoriasis Data

The results for all 3 comparisons were presented in Table 4A. The positive effect of batch adjustment on Meta-TDGR's performance is striking and consistent across all comparisons and datasets. When the data were adjusted for batch effect before classification, TGDR and Meta-TGDR had identical miss-classification rates on training and testing samples in all 3 comparisons and TGDR slightly outperformed in terms of GBS.

**Table 4:** Performance of Classifiers for Psoriasis data.

*A. Binary Classifiers*							
		Training (n = 360)				Test set (n = 89)	
	Method	# genes	Error (%)	5-fold CV (%)	GBS	PE (%)	GBS
**LS vs Normal** Training: 233 Test: 49	TDGR (adjusted)	30	0	0.86	0.0001	0	0.0006
	Meta-TGDR (unadjusted)	18	0	0.86	0.0010	2.04	0.0084
	Meta-TGDR (adjusted)	22	0	0.86	0.0006	0	0.0028
	TDGR w/Bagging (adjusted, BF >30%)	18	0	–	0.0011	0	0.0004
	Meta-TGDR w/Bagging (adjusted, BF >30%)	10	0	–	0.0012	0	0.0032
**LS vs NL** Training: 271 Test: 68	TDGR (adjusted)	35	0	1.48	0.0009	1.47	0.0136
	Meta-TGDR (unadjusted)	26	1.11	1.85	0.0105	2.94	0.0294
	Meta-TGDR (adjusted)	25	0	1.48	0.0036	1.47	0.0143
	TDGR w/Bagging (adjusted, BF >30%)	22	0	–	0.0021	1.47	0.0144
	Meta-TGDR w/Bagging (adjusted, BF >40%)	16	1.48	–	0.0041	1.47	0.0142
**NL vs Normal **Training: 216 Test: 61	TDGR (adjusted)	26	0	0	1.5×10^−5^	0	7.3×10^−5^
	Meta-TGDR (unadjusted)	40	5.56	18.06	0.0570	8.20	0.0659
	Meta-TGDR (adjusted)	22	0	1.85	0.0032	0	0.0054
	TDGR w/Bagging (adjusted, BF >30%)	24	0	–	2.4×10^−5^	0	7.3×10^-5^
	Meta-TGDR w/Bagging (adjusted, BF >40%)	21	0	–	0.0033	0	0.0054

*B. Multiclass Classifiers*							
Method		# genes	Error (%)	5-fold CV (%)	GBS	PE (%)	GBS
Pairwise-coupling		76	0	1.67	0.0067	0	0.0013
Multi-TDGR	Adjusted	60	0	1.67	0.0144	0	0.0013
	Bagging (BF >40%)	39	0	–	0.0052	0	0.0006
Multi-Meta-TGDR	Unadjusted	5	18.33	21.39	0.1734	26.97	0.1629
	Adjusted	13	7.77	8.61	0.1296	6.74	0.0393

A. Comparison between TGDR and Meta-TGDR for binary classifiers. B. Comparisons between TGDR and Meta-TGDR for 3-class classifiers.

***LS versus normal:*** Using TGDR on batch-adjusted expression from **LS and Normal** skin samples, we identified 30 biomarkers with a 0% and 0.86% training and CV-5 error, respectively. Bragging (N_B_ = 100) frequencies were above 5% for all 30 genes. By considering a series of cutoff values for the frequency (5%–50%), a 30% for BF was chosen as it minimized the GBS and misclassification rates with the smallest number of non-zero genes leading to a final model with 18 genes (Table 4). Meta-TGDR (after batch adjustment) identified 22 biomarkers all with BF>5%. Cut-off for BF was set at 40% and among the 10 selected genes (see Table 5), 6 (p<0.0001) overlapped with the 18 genes in the TGDR bagging classifier.

**Table 5:** Psoriasis LS versus Normal genes by TGDR and Meta-TGDR after Bagging.

			TGDR		Meta-	TGDR	
Probe	Symbol	Description	β	β^Yao^	β^Gud^	β^SF+^	β
229963_at	BEX5	brain expressed, X-linked 5	−0.2958				
207356_at	DEFB4A	defensin, beta 4A	1.9258	1.3188	2.1196	1.6617	1.9405
224209_s_at	GDA	guanine deaminase	0.8995	1.3512	1.4587	1.1601	1.5021
202411_at	IFI27	interferon, alpha-inducible protein 27	0.69	0.0313	0.083	0.0556	0.2784
1555745_a_at	LYZ	lysozyme	0.312	0.1929	0.0934	0.1837	0.0633
205916_at	S100A7	S100 calcium binding protein A7	0.6612	0.2597	0.414	0.2862	0.4206
212492_s_at	KDM4B	lysine (K)-specific demethylase 4B	−0.1272				
201846_s_at	RYBP	RING1 and YY1 binding protein	−1.4184	−0.3202	−0.1995	−0.4103	−0.2907
201416_at	SOX4	SRY (sex determining region Y)-box 4	−0.1703				
215363_x_at	FOLH1	folate hydrolase (prostate-specific membrane antigen) 1	0.3342				
203335_at	PHYH	phytanoyl-CoA 2-hydroxylase	−0.3569				
205758_at	CD8A	CD8a molecule	0.1235				
1556069_s_at	HIF3A	hypoxia inducible factor 3, alpha subunit	0.2577				
213424_at	KIAA0895	KIAA0895	−0.3158				
205132_at	ACTC1	actin, alpha, cardiac muscle 1	−0.1815				
1431_at	CYP2E1	cytochrome P450, family 2, subfamily E, polypeptide 1	0.2671				
230005_at	SVIP	small VCP/p97-interacting protein	−0.1723				
202668_at	EFNB2	ephrin-B2	−0.1202				
205471_s_at	DACH1	dachshund homolog 1 (Drosophila)		−0.1171	−0.0807	−0.0722	−0.0721
229625_at	GBP5	guanylate binding protein 5		0.1256	0.0786	0.0659	0.1468
213293_s_at	TRIM22	tripartite motif containing 22		0.1796	0.1428	0.1477	0.0042
202267_at	LAMC2	laminin, gamma 2		−0.0785	−0.0844	−0.0971	−0.0942

Here, Normal skin samples serve as the reference. Bagging frequency >30% for TGDR and >40% for Meta-TGDR.

***LS versus NL:*** TGDR signature for **LS vs NL** classification included 35 biomarkers with a training error of 0% and 1.48% in 5-CV. Applying Bagging procedure (N_B_ = 100) the final model (with BF >30%) included 22 genes (see Table 6). Meta-TGDR on the adjusted data identified a 25 genes signature, all with BF >5% and 16 of them above the selected cut-off of 40% for BF (See Table 6). There is still an impressive overlapping (n = 11) between these 16 genes and the 22 genes chosen by TGDR bagging model (Fisher's test: p<0.0001).

**Table 6:** Psoriasis LS versus NL genes by TGDR and Meta-TGDR after Bagging.

			TGDR		Meta-	TGDR	
Probe	Symbol	Description	β	β^Yao^	β^Gud^	β^SF+^	β
210002_at	GATA6	GATA binding protein 6	−0.1895				
235603_at	HNRNPU	heterogeneous nuclear ribonucleoprotein U (scaffold attachment factor A)	−0.7306	−0.4787	−0.4382	−0.5031	−0.694
231875_at	KIF21A	kinesin family member 21A	−0.1396				
233819_s_at	LTN1	listerin E3 ubiquitin protein ligase 1	−0.0771				
203476_at	TPBG	trophoblast glycoprotein	0.4798	0.1412	0.2202	0.2286	0.5236
234335_s_at	FAM84A	family with sequence similarity 84, member A	−0.1498				
230828_at	GRAMD2	GRAM domain containing 2	−0.4782	−0.0935	−0.1705	−0.306	−0.1539
224171_at	LSM14B	LSM14B, SCD6 homolog B (S. cerevisiae)	−0.2053				
230699_at	PGLS	6-phosphogluconolactonase	−0.5561	−0.0144	−0.2076	−0.328	−0.1485
1552797_s_at	PROM2	prominin 2	−0.1398				
226404_at	RBM39	RNA binding motif protein 39	−0.0434	−0.1364	−0.155	−0.1385	−0.2417
202648_at	RPS19	ribosomal protein S19	−0.4381	−0.3294	−0.6499	−0.9706	−0.7572
230586_s_at	ZNF703	zinc finger protein 703	−0.76	−0.1284	−0.3829	−0.2787	−0.9174
211661_x_at	PTAFR	platelet-activating factor receptor	0.7216				
203335_at	PHYH	phytanoyl-CoA 2-hydroxylase	−0.3971				
213849_s_at	PPP2R2B	protein phosphatase 2, regulatory subunit B, beta	−0.1023				
226367_at	KDM5A	lysine (K)-specific demethylase 5A	−0.3457	−0.0369	−0.0484	−0.0447	−0.0001
228132_at	ABLIM2	actin binding LIM protein family, member 2	−0.5047	−0.5503	−0.5072	−0.5825	−0.7550
202267_at	LAMC2	laminin, gamma 2	−0.1007	−0.0953	−0.0976	−0.1271	−0.0387
213424_at	KIAA0895	KIAA0895	−0.2031				
205132_at	ACTC1	actin, alpha, cardiac muscle 1	−0.2045				
203127_s_at	SPTLC2	serine palmitoyltransferase, long chain base subunit 2	1.3511	0.4363	0.665	0.7786	0.9813
201487_at	CTSC	cathepsin C		0.0171	0.0155	0.0174	0.0000
217388_s_at	KYNU	Kynureninase		0.1287	0.1755	0.1958	0.0002
205863_at	S100A12	S100 calcium binding protein A12		0.3597	0.5641	0.5861	0.0486
243417_at	ZADH2	zinc binding alcohol dehydrogenase domain containing 2		−0.1611	−0.1703	−0.1686	−0.1063
211661_x_at	PTAFR	platelet-activating factor receptor		0.6241	0.6422	0.7737	0.9329

Non-lesional skin samples serve as the reference. Bagging frequency >30% for TGDR and >40% for Meta-TGDR.

Although Meta-TGDR is more parsimonious, k (the number of steps\iterations) is always dramatically bigger than in TGDR for the same value of the tuning parameter τ in both algorithms. One possible explanation is that by allowing different coefficients for a specific gene across studies, the direction of updating path in individual study may differ, probably leading to a cancel-out among one another. Therefore, the gradients might descend at higher speed in TGDR than meta-gradients in Meta-TGDR and the maximized likelihood value might be reached within fewer steps. Since separate gradient matrixes were calculated for each study in meta-TGDR and the tuning parameter k is always bigger (gradient matrix and threshold function must be calculated in each iteration), the computing time of meta-TGDR is expected to be substantially longer than that of TGDR (e.g., for LS vs NL comparison, meta-TGDR used 9 minutes and 42 seconds to determine on the tuning parameters and to estimate the coefficients while TGDR used 2 minutes and 46 seconds).

#### Psoriasis 3-class classification

Here we evaluate the performance of the multi-TGDR versus the classifier build using all pairwise binary TGDRs. The performance of the Meta-multi-TGDR for adjusted and unadjusted data is also presented.

Using multi-TGDR on the training set, we identified 60 genes with 0% training error 1.67% error in 5 fold CV. Interestingly; the number of selected genes by multi-TGDR is approximately the sum of all individualized binary TGDRs. The size went down to 39 genes (Table 6) when bagging frequency being larger than the selected cut-off (40%). Again, disposal of the low-BF genes did not hurt the predictive performance. On the contrary, it improves the predictive performance in terms of GBS for both training and testing samples.

**Figure 1:**
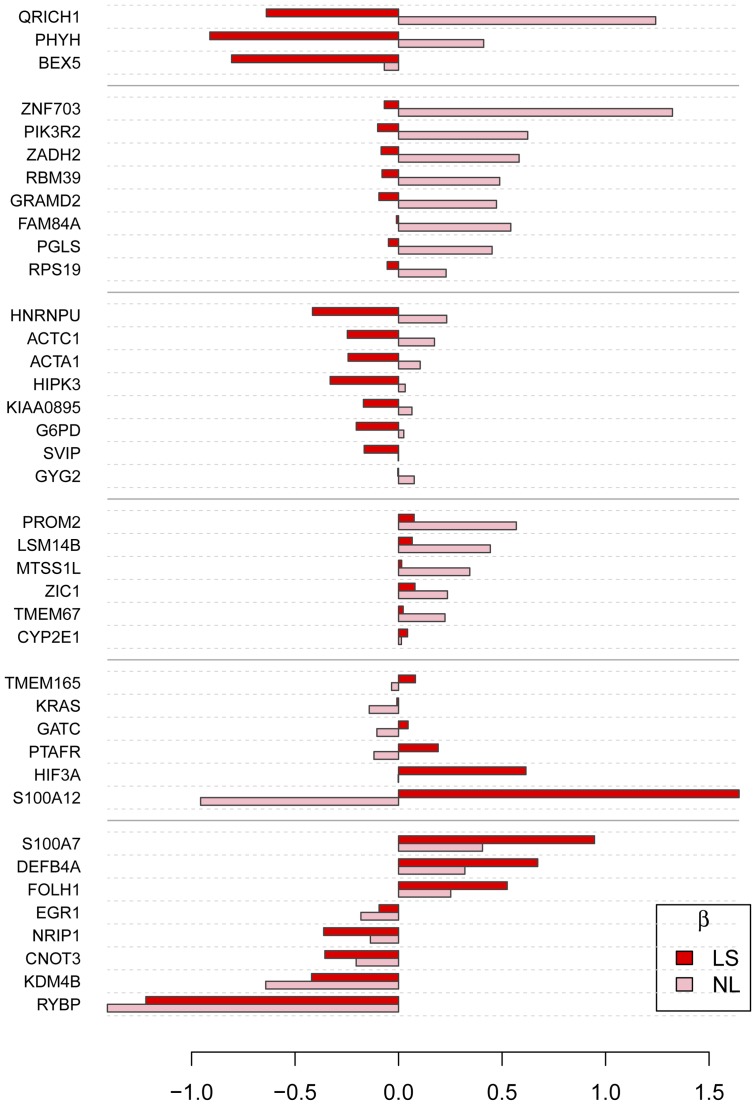
The estimated coefficients of the genes selected by multi-TGDR in the psoriasis data. Normal skin tissues from controls served as the reference. NL: Non-Lesional skin; LS: Lesional skin.

The classifier built by combining the 3 pairwise binary TGDR had the same in-training performance as multi-TGDR using 76 genes, with 44 of them being part of the multi-TGDR classifier (p<0.0001). The inconsistency between two algorithms is partially because local optimal points in individualized binary TGDRs cannot warrant the global optimality in the multi-TGDR. With this data set, multi-TGDR and pairwise binary TGDR had similar performance while multi-TGDR was more parsimonious. Additionally, total computing time was about 8 minutes for pair-wise strategy and 4.5 minutes for the multi-TGDR framework. Multi-TGDR with Bagging provided the best performance (see Table 7 and Figure 1). This shed some evidence on appropriation and accuracy of the multi-TGDR framework. Certainly, further evaluation using independent test sets is needed.

**Table 7:** Psoriasis 3 classes genes selected by multi-TGDR after Bagging.

Probe	Symbol	Description	β_NL	β_LS	Freq
203872_at	ACTA1	actin, alpha 1, skeletal muscle	0.1043	−0.2438	0.51
229963_at	BEX5	brain expressed, X-linked 5	−0.0688	−0.8066	0.75
207356_at	DEFB4A	defensin, beta 4A	0.3202	0.672	0.46
235603_at	HNRNPU	heterogeneous nuclear ribonucleoprotein U (scaffold attachment factor A)	0.2325	−0.4157	0.68
205863_at	S100A12	S100 calcium binding protein A12	−0.9565	1.6449	0.42
205916_at	S100A7	S100 calcium binding protein A7	0.4054	0.9465	0.81
226825_s_at	TMEM165	transmembrane protein 165	−0.0338	0.0811	0.58
206373_at	ZIC1	Zic family member 1	0.2361	0.0792	0.59
203239_s_at	CNOT3	CCR4-NOT transcription complex, subunit 3	−0.2049	−0.3561	0.43
201693_s_at	EGR1	early growth response 1	−0.182	−0.094	0.47
234335_s_at	FAM84A	family with sequence similarity 84, member A	0.542	−0.0105	0.99
214711_at	GATC	glutamyl-tRNA (Gln) amidotransferase, subunit C homolog (bacterial)	−0.1048	0.0458	0.45
230828_at	GRAMD2	GRAM domain containing 2	0.4728	−0.0949	0.85
207764_s_at	HIPK3	homeodomain interacting protein kinase 3	0.032	−0.3303	0.54
212492_s_at	KDM4B	lysine (K)-specific demethylase 4B	−0.6416	−0.4202	0.88
214352_s_at	KRAS	v-Ki-ras2 Kirsten rat sarcoma viral oncogene homolog	−0.1416	−0.0083	0.55
224171_at	LSM14B	LSM14B, SCD6 homolog B (S. cerevisiae)	0.443	0.0658	0.93
1556175_at	MTSS1L	metastasis suppressor 1-like	0.3438	0.015	0.85
202600_s_at	NRIP1	nuclear receptor interacting protein 1	−0.1361	−0.3617	0.61
230699_at	PGLS	6-phosphogluconolactonase	0.4517	−0.049	0.79
229392_s_at	PIK3R2	phosphoinositide-3-kinase, regulatory subunit 2 (beta)	0.6238	−0.101	0.78
1552797_s_at	PROM2	prominin 2	0.5688	0.0752	0.84
229806_at	QRICH1	glutamine-rich 1	1.2418	−0.6387	0.86
226404_at	RBM39	RNA binding motif protein 39	0.4885	−0.0797	0.96
202648_at	RPS19	ribosomal protein S19	0.2297	−0.0548	0.9
201846_s_at	RYBP	RING1 and YY1 binding protein	−1.4064	−1.2206	0.99
1563646_a_at	TMEM67	transmembrane protein 67	0.2242	0.0222	0.48
243417_at	ZADH2	zinc binding alcohol dehydrogenase domain containing 2	0.5824	−0.0841	0.92
230586_s_at	ZNF703	zinc finger protein 703	1.3228	−0.0688	0.99
215363_x_at	FOLH1	folate hydrolase (prostate-specific membrane antigen) 1	0.2521	0.5247	0.71
211661_x_at	PTAFR	platelet-activating factor receptor	−0.1189	0.1913	0.51
203335_at	PHYH	phytanoyl-CoA 2-hydroxylase	0.4112	−0.9118	0.61
202275_at	G6PD	glucose-6-phosphate dehydrogenase	0.0251	−0.2045	0.52
1556069_s_at	HIF3A	hypoxia inducible factor 3, alpha subunit	−0.0016	0.6154	0.62
213424_at	KIAA0895	KIAA0895	0.0646	−0.1698	0.48
215695_s_at	GYG2	glycogenin 2	0.0756	−0.0037	0.55
205132_at	ACTC1	actin, alpha, cardiac muscle 1	0.1735	−0.2476	0.6
1431_at	CYP2E1	cytochrome P450, family 2, subfamily E, polypeptide 1	0.0139	0.0433	0.45
230005_at	SVIP	small VCP/p97-interacting protein	−0.0019	−0.1661	0.43

There are 39 genes in the final model. Normal tissue from healthy controls serves as the reference. Bagging frequency >40%.

Surprisingly, the performance of Meta-multi-TGDR, where coefficients for both classes and studies are included is not impressive. This may partially due to the fact that Meta-multi-TGDR intends to find consistent-expressed genes across all classes and studies (one possible reason why the number of non-zero genes in multi-Meta-TGDR is the smallest). Based on the analyses conducted here, we illustrated that TGDR on the adjusted data has a similar or better performance compared to Meta-TGDR, thus we think Meta-multi-TGDR, with its increase complexity and computing burden, is quite unnecessary. Nonetheless, the Meta-multi-TGDR greatly improved after batch adjustment reducing training error from 18.33% to 7.11% and predictive error (on test set) from 27% to 6.74%, demonstrating that the adjustment of batch effect is imperative.

## Discussion

When several microarray studies address the same or similar objectives, it is statistically more robust to carry out the analysis by pooling all studies together. To identify molecular signatures that discriminate among different disease status or stages on the pooled data, one can either apply TGDR to the batch-effect adjusted expression values for all samples, or use Meta-TGDR to select consistently informative genes and obtain the overall estimates using the procedure we proposed in the paper.

Using real-world applications, we showed that TGDR and Meta-TGDR have approximately equal predictive performance when the data has been adjusted for batch-effect. Compared to the latter method, TGDR on the adjusted data saves computing time, and do not require that all classes must be represented in each study. However, the stability of Meta-TGDR is usually better than TGDR as shown by the analyses of psoriasis data, and future work must be done to improve more on stability of TGDR. Nonetheless, applying Meta-TGDR on the unadjusted data had worse predictive performance compared to the analyses on the adjusted data. This verified our conjecture that Meta-TGDR aims mainly at selecting consistent genes across studies, with few to no capacity to adjust for a large batch-effect.

Additionally, we assembled our analyses with the Bagging procedure [12]. The benefits of Bagging including improved selection stability; more classification accuracy; and protection against over-fitting are clearly illustrated here.

Since multi-TGDR is an extension to binary TGDR, whose performance had been proved to be equal or superior to many other classification methods in the original papers [9], we did not compare the multi-TGDR with other classification methods and rather focus on the important issues addressed here: making Meta-TGDR useful in practice by offering a solution to the prediction of independent datasets, comparing TGDR and Meta-TGDR performance after batch adjustment and extending both algorithms to the multiclass setting. Future work should involve a comprehensive comparison of multi-TGDR' performance with other multi-class classification methods and the identification of data types most suitable for multi-TGDR. We currently devote ourselves to such extensive and laborious work. Additionally, as the numbers of classes in the applications presented here are not big (4 in LC and 3 in psoriasis data, respectively), future work will include some applications of the multi-TGDR framework with a large number of classes, where the performance of pair-coupling has been reported to decrease dramatically, to see if a single likelihood-based classifier like multi-TGDR can be a rescue.
